# Occurrence of radiopaque and mixed lesions at periapical region in patients with rheumatoid arthritis and ankylosing spondylitis: a retrospective study

**DOI:** 10.1186/s12903-023-03493-y

**Published:** 2023-10-24

**Authors:** Melis Yilmaz, Fatma Tunc

**Affiliations:** 1 Faculty of Dentistry, Department of Endodontics, Mersin University, Mersin, Turkey; 2 Private Practice, Mersin, Turkey; 3 Faculty of Dentistry, Department of Endodontics, Gaziantep University, Gaziantep, Turkey

**Keywords:** Rheumatoid arthritis, Ankylosing spondylitis, Radiopaque lesion, Mixed lesion

## Abstract

**Background:**

Rheumatoid arthritis (RA) and ankylosing spondylitis (AS) have different effects on bones, cartilage and joints, sometimes destroying the spine and joints, and other times causing new bone formation. This study aimed to evaluate the effects of RA and AS on the types (radiolucent, radiopaque and mixed) of periapical lesions in jaw bones.

**Methods:**

This study included 708 individuals (97 with AS, 327 with RA and 284 healthy controls (C)) and a total of 17,118 teeth (AS: 2,442; RA: 7,638; C: 7,038). The number of teeth, extracted teeth and teeth with root canal treatment and the presence of radiopaque, radiolucent and mixed periapical lesions were recorded from dental panoramic radiographs. Kruskal–Wallis and chi-square tests were used for statistical analysis.

**Results:**

The frequency of radiopaque lesions in the AS and RA groups was similar (p > 0.05) and significantly higher than in the C group (p < 0.05) (AS: 13.4%; RA: 6.1%; C: 2%). Mixed lesions (AS: 3.1%; RA: 4.0%; C: 0.4%) were statistically significantly higher for the RA group compared to the C group (p < 0.05), while the AS–C and AS–RA groups were similar (p > 0.05). There was no significant difference in terms of radiolucent lesions among groups (p > 0.05).

**Conclusion:**

Radiopaque apical lesions were frequent in RA and AS patients, while mixed lesions were significantly higher in RA patients.

## Background

Rheumatoid arthritis (RA) and ankylosing spondylitis (AS) are genetically transmitted, common, severe and chronic inflammatory skeletal diseases. Both diseases are known to cause disability and reduced life quality [1–3], and they follow a similar clinical course in terms of symptoms, radiographic characteristics and serological testing [4]. However, these diseases have different treatment plans, and the drugs used in both diseases have different effects on patients [2, 5–7].

The effects of these two diseases on bones, cartilage and joints differ. In patients with RA, excessive destruction of the joint is highly pronounced, and it is rare to observe tissue repair. In AS patients, the destruction of the spine and joints leads to localised ankylosis through the formation of new bone in the area [8]. Such effects on jaw bones and the development of periapical lesions have not been mentioned in the literature.

The radiographic evaluation of jaw bones is an integral part of routine dental examinations and endodontic treatment. To date, radiolucent and radiopaque lesions identified in the periradicular region have generally been checked upon radiological evaluation as part of diagnostic procedures. Detected lesions indicate the need for further investigation to determine the condition of that tooth [9–11]. However, mixed lytic and sclerotic periapical and jaw bone lesions are also frequently encountered [12, 13]. Additionally, radiopaque lesions and lesions of varied density are less frequent than radiolucent lesions [13].

The literature has established an association between exposure of the head–neck area to high-dose radiation, and systemic conditions, including diabetes, cardiovascular disease, osteoporosis, inflammatory bowel disease (IBD), inherited coagulopathies, biological medications [14, 15], low birth weight, physical fitness, smoking and periapical lesions [15]. Despite the known effects of RA and AS on the bones, cartilage and joints [8] and their adverse effects on periodontal [16] and temporomandibular joint health [17], few studies have investigated the impact of both diseases on the development of periapical pathologies [18–20]. Additionally, no lesion type was mentioned (other than radiolucent lesions) in previous studies [18–20]. In cases presenting radiopaque and mixed lesions, an assessment should be undertaken to determine whether they are linked to the jaw and teeth where they are localised [10]. If an odontogenic sclerotic lesion has been identified, steps should be taken to establish whether a dental infection is the cause. Both types of lesions are observed in hereditary and developmental disorders, osteomyelitis, benign/malignant primary bone tumours and metastases [13]. Cure et al. reported the findings of such mixed lesions in patients with cancer in forms such as osteoradionecrosis, bisphosphonate-induced osteoradionecrosis and mandibular osteomyelitis, as well as in patients with ossifying fibroma, fibrous dysplasia and renal osteodystrophy, albeit of varying sizes in the latter cases [12]. To the best of the authors’ knowledge, no study has evaluated the association between AS and RA and radiopaque/mixed periapical lesions. However, the literature shows that periapical cemento-osseous dysplasia (PCOD) may occur in the jaws as different types of lesions, as investigated in this study. This dysplasia can be identified by three stages according to the radiographic image: (1) the radiolucent (osteolytic) stage; (2) the radiolucent–radiopaque stage, which is characterised by a dense radiopaque mass in the centre; and (3) the radiopaque stage (surrounded by a thin radiolucent area) [13].

The objective of the present study was to evaluate the frequency of radiopaque, radiolucent and mixed lesions in patients with AS and RA.

## Materials and methods

The present retrospective study was performed in the Departments of Endodontics at Gaziantep University Faculty of Dentistry and Mersin University Faculty of Dentistry after approval by the Gaziantep University Clinical Research Ethics Committee (Approved 24.02.2021, No. 2021/45). To predict the number of samples, a power analysis using G*Power 3.1.9.4 software was performed. The difference between apical lesion incidence rates was taken as Δ: 32%, and the number of samples determined for Power: 0.80 and α: 0.05 was determined as a minimum of n:41 individuals for each group. As a result of the Cohen’s Kappa examination, the inter-observer agreement was 0.8541.

Prior to this study, patient records from the Gaziantep University Faculty of Dentistry (2015–2020) and Mersin University Faculty of Dentistry (2017–2020) were scanned to identify all patients with AS and RA, resulting in 138 AS and 500 RA patients (638 in total). Patients with diabetes, hypertension, hyperthyroidism, corticosteroid medication, cancers, a history of transplantation, connective tissue diseases (systemic lupus erythematosus and Sjogren’s syndrome, etc.), those taking bisphosphonates for osteoporosis patients, those younger than age 18 and older than age 80 and those with fewer than six teeth were excluded from the study. Ultimately, 424 eligible patients (97 with AS and 327 with RA) were included in the study group. The healthy control (C) group included 284 healthy subjects without any systemic conditions and with more than six teeth. Dental digital panoramics (DPRs) of all subjects were examined to record the number of teeth, extracted teeth and root canaltreated (RCT) teeth. The presence of radiopaque, radiolucent and mixed lesions was recorded in all patients, with 2,442 in the AS group, 7,638 in the RA group and 7,038 in the C group (17,118 in total). Apical status was assessed using the periapical index (PAI) score, according to which five scores were attributed to the apical area of the radiographic images as follows: (1) normal periapical structures; (2) small changes in bone structure; (3) changes in the bone structure with little mineral loss; (4) periodontitis with a well-defined radiolucent area; and (5) severe periodontitis with exacerbating features. For multirooted teeth, the values of the root with the highest PAI score were recorded. Teeth scoring 3, 4 or 5 were noted as having apical periodontitis [21].

A radiographic evaluation was performed by two different endodontists simultaneously. Each observer carefully reviewed the findings and confirmed them independently. In cases of disagreement between the observers, a dental and maxillofacial radiologist was consulted as a third observer to achieve observer agreement.

### Statistical analysis

Continuous variables were summarised as mean ± standard deviation or median (min–max) according to the normality assumption. Categorical variables were presented as numbers and percentages. The normal distribution control of the variables of the evaluated parameters was done with the Shapiro–Wilk test. In the comparison of more than two groups, the Kruskal–Wallis test was used, and the Dunn test was used as a *post hoc* test in cases of significance. A chi-square test was used to investigate the correlation between categorical variables. The identification of any significant correlation was followed by two comparison ratios. Multiple logistic regression models were built to adjust the effects of possible confounding factors on the main outcomes. Variance inflation factors (VIFs) were calculated to evaluate the issue of multicollinearity. Adjusted odds ratios (ORs) and 95% confidence intervals (CIs) were estimated.

## Results

**Fig. 1:**
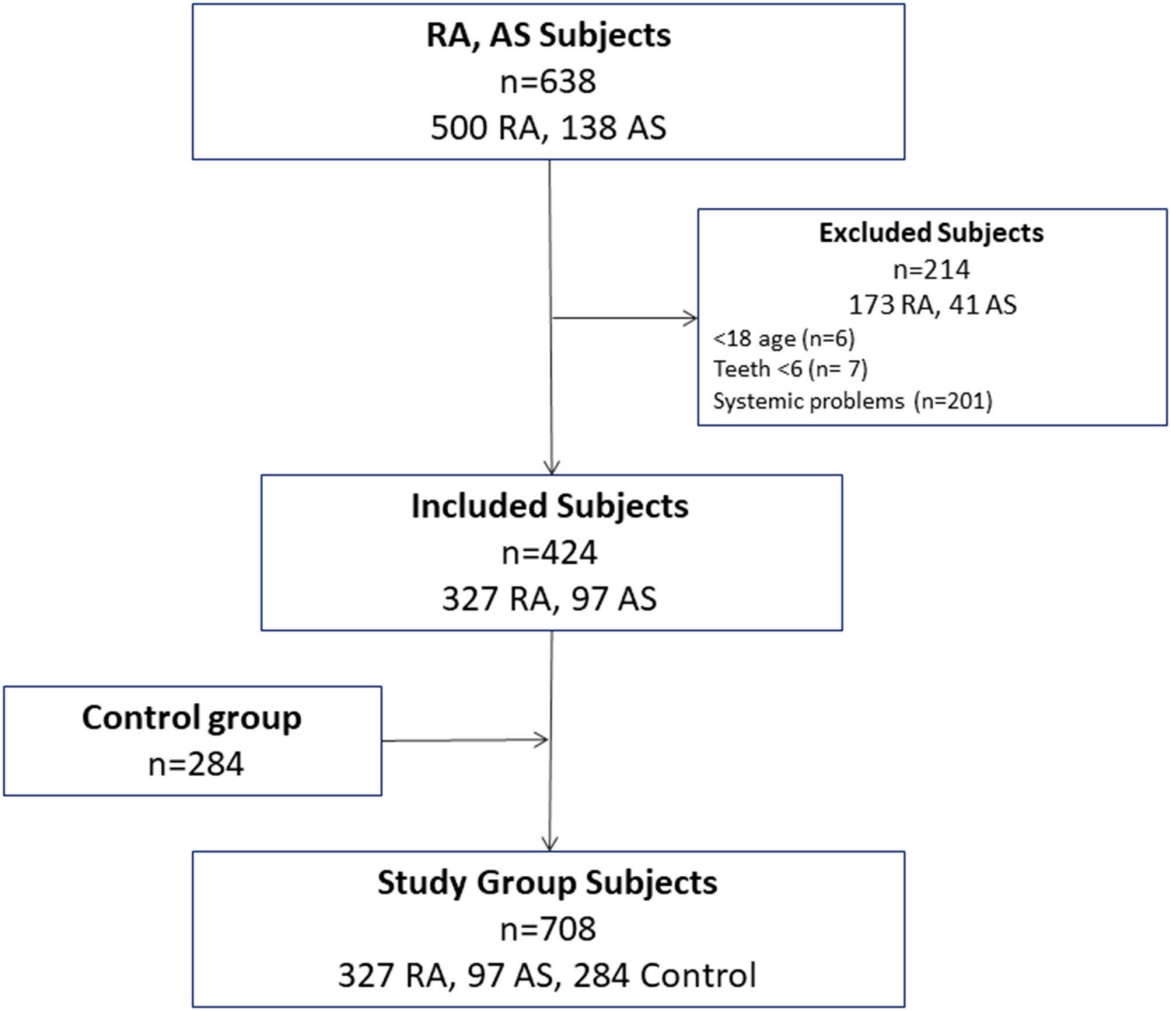
Flowchart of the subjects

This study included 97 AS patients, 327 RA patients (two study groups) and 284 healthy individuals (the C group) (Fig. 1). The number of subjects in each group and the distribution of age, gender, number of teeth, extracted teeth and teeth with RCT are presented in Table 1. The number of radiopaque, radiolucent and mixed lesions in all patients are shown in Table 2. There was a significant difference among the groups in terms of gender, age and the total number of teeth present. Therefore, multivariate binary logistic regression models were built to adjust the effect of possible confounding factors (Table 1) on the main outcomes (Table 3). The VIFs ranged from 1 to 1.4. According to the adjusted ORs and *p*-values, there was no significant difference among the groups in terms of the findings of radiolucent lesions. Following adjustments for the effects of age, gender and number of teeth present, the rate of occurrence of radiopaque lesions was found to be 6.67 times higher (OR = 6.67, 95% CI = 2.35–18.91, *p* = 0.001) in the AS group and 2.9 times higher (OR = 2.93, 95% CI = 1.13–7.91, *p* = 0.001) in the RA group compared to the C group. In terms of the occurrence of mixed lesions, the risk was 11.86 times higher in the RA group than in the C group, and there were marginally significant odds for the AS group (OR = 8.93, 95% CI = 0.91–87.27, *p* = 0.060) compared to the C group.

**Table 1:** The number of subjects in the groups; distribution of age, gender, number of teeth, extracted teeth and teeth with RCT

		Ankylosing Spondylitis	Rheumatoid Arthritis	Control	P
Subjects n(%)		97(%13.7)	327(%46.2)	284 (%40.1)	
Age ( mean+_ SD)		41.84 ± 11.18	46.55 ± 13.75*	43.15 ± 15.69	0.002
Gender	Male	48(%49.5)	82 (%25.1)*	110(%38.7)	< 0.001
	Female	49(%50.5)	245(%74.9)*	174 (%61.3)	
Number of teeth, median[min.-max.]		26 [12–28]	25 [7–28]*	26 [6–28]	< 0.001
Extracted teeth, median[min.-max.]		2 [0–16]	3 [0–21]*	2 [0–22]	< 0.001
Teeth with RCT, median[min.-max.]		1[0–6]	0 [0–12]	0 [0–10]	0.212

*RA group is statisticaly different from the AS and control group in age, gender, teeth number and exracted teeth numbers

**Table 2:** Distribution of all parameters to the groups with the number of subjects

		Ankylosing Spondylitis n(%)	Rheumatoid Arthritis n(%)	Control n(%)	Total n(%)	P
Radiolucent lesion	absent	69 (%71.1)	227 (%69.4)	209 (%73.6)	505 (%71.3)	0.523
	present	28 (%28.9)	100 (%30.6)	75 (%26.4)	203 (%28.7)	
Radiopaque lesion	absent	84 (%86.6)_a_	307(%93.9) _a_	278 (%97.9) _b_	669 (%94.5)	< 0.001
	present	13 (%13.4)_a_	20 (%6.1) _a_	6 (%2.1) _b_	39 (%5.5)	
Mixed lesion	absent	94 (%96.9)_a, b_	314 (%96.0)_b_	283 (%99.6) _a_	691(%97.6)	0.013
	present	3 (%3.1) _a, b_	13 (%4.0) _b_	1 (%0.4) _a_	17 (%2.4)	

*Different letters show the statistical significance. Bonferroni correction was made for comparison of two ratios

**Table 3:** Multivariate binary logistic regression analysis results for radiolucent, radiopaque and mixed lesions

	Radiolucent lesion		Radiopaque lesion		Mixed lesion	
Variable	OR[95% CI]	p	OR[95% CI]	p	OR[95% CI]	p
As vs. control	1.03 [0.57–1.85 ]	0.926	6.67 [2.35–18.91 ]	0.001*	8.93 [0.91–87.27 ]	0.060
RA vs. control	1.25 [0.84–1.87 ]	0.274	2.93 [1.13–7.61 ]	0.027*	11.86 [1.52–92.29 ]	0.018*
Female vs. Male	0.77 [0.52–1.13 ]	0.184	3.04 [1.2–7.7 ]	0.019*	0.76 [0.27–2.15 ]	0.607
Age	1.01 [0.99–1.02 ]	0.266	1.01 [0.98–1.04 ]	0.663	1.01 [0.97–1.06 ]	0.591
Number of teeth	0.99 [0.94–1.03 ]	0.524	1.26 [1.06–1.48 ]	0.007	1.01 [0.89–1.14 ]	0.910

*Significant at 0.05 level, OR: Adjusted ORs, CI: Confidence interval

The statistical analyses did not reveal any significant difference among the RA, AS and C groups in radiolucent lesions (p > 0.05). When the distribution of individuals with radiopaque lesions was examined, patients with RA and AS were more likely to have these lesions than the C group. The difference was statistically significant (p < 0.05). However, the distribution of mixed lesions provided a different finding than the distribution of radiopaque lesions. The mixed lesions, in comparison with the C group, were found at a significantly higher rate among patients with RA (p < 0.05) and at a similar number among patients with AS (p > 0.05).

**Fig. 2:**
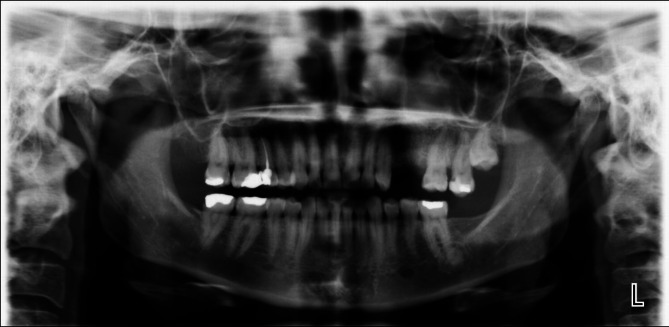
A radiopaque lesion associated with the left lower first molar in an AS patient

According to the radiological data in this study, the distribution of radiopaque lesions (Fig. 2) and mixed lesions by tooth and jaw of localisation was as follows:

- In the AS group, 13 patients had radiopaque lesions in 16 teeth that were all localised in the mandible. Their distribution was as follows: two incisors, six premolars and eight molars. Three AS patients, each with one mandibular molar, had mixed lesions.
- In the RA group, 20 patients had radiopaque lesions in 21 teeth. Only one of these lesions was localised in the maxilla, and the remaining lesions were in the mandible. Their distribution was as follows: one maxillary incisor, nine mandibular premolars and 11 mandibular molars. Conversely, 16 mixed lesions were detected in 13 patients, with all localised in the mandible. Four were in premolars, and twelve were in molars.
- In the C group, radiopaque lesions were identified in six patients, with all localised in the mandible. Three were in premolars, and three were in molars. Only one patient had a mixed lesion localised in a mandibular molar.

**Fig. 3:**
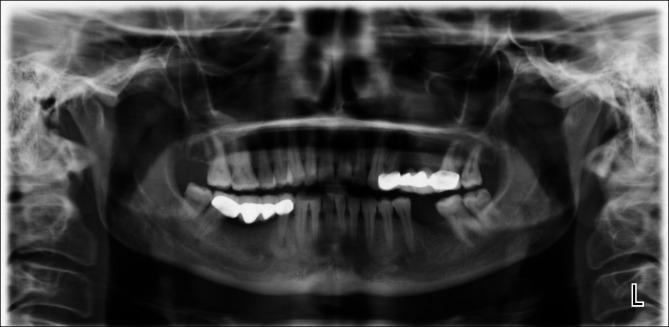
A mixed lesion associated with the right lower first premolar in an RA patient

Although the sclerotic and lytic areas in the observed mixed lesions varied in size, all were characterised by the presence of centrally localised radiolucent areas and peripheral radiopaque areas (Fig. 3).

## Discussion

The literature points out a large number of diseases that cause radiolucent and radiopaque lesions of jaw bones [9–12]. The current study is the only one that evaluates the presence of radiolucent, radiopaque and mixed lesions. According to the findings, AS and RA may be added to the list of diseases affecting the formation of radiopaque and mixed lesions or associated with these types of lesions. Nevertheless, it should be noted that radiolucent, radiopaque and mixed lesions can be seen in both diseases. In addition, patients with RA and AS were found to have more radiopaque lesions than the C group, and this difference was statistically significant (p < 0.05). Mixed lesions were only more common in the RA group than in the C group (p < 0.05).

Interestingly, radiopaque lesions were more common in the RA and AS groups than in the C group, while mixed lesions were only more common in the RA group than in the C group. RA is considered an autoimmune disease in which synovial tissues develop destructive pannus, leading to erosion and loss of joint function. In contrast, AS is an autoimmune disease that is less likely to cause classical tissue destruction and is sometimes associated with enough new cartilage and bone to cause ankyloses [8]. The mechanisms affecting the authors’ findings may be similar to those that cause certain levels of tissue repair in bones in RA and those that cause spinal ankylosis in AS, as Lories et al. mentioned [8].

Apical lesions have been detected using DPRs, periapical radiographs and cone beam computed tomography [15]. The DPRs of the RA and AS patients studied were in the data systems. Therefore, the study was designed as retrospective, and apical lesions were detected from DPRs to save patients from unnecessary radiation. However, detecting apical lesions from a panoramic view, especially in the anterior areas of the jaw, creates limitations. In radiographs, all types of jaw bone lesions can indicate malignancy, and malignant lesions have ill-defined borders [22]. The borders of all lesions detected in this study were clear. Therefore, they were not evaluated as malignant lesions. In a study evaluating diseases with mixed lesions, including PCOD, mixed lesions were sampled as radiolucencies with centrally localised sclerotic areas [13]. However, the mixed lesions observed in the current study were radiopaque in the periphery and radiolucent in the centre. Therefore, the hypothesis is that the mechanism of the development of mixed lesions in patients with RA and AS differs from other diseases with these lesions [13]. Furthermore, the majority of these lesions were detected in the mandible, similar to PCOD. This may suggest that the mixed lesions detected in RA and AS patients may be similar to those seen in PCOD. However, it has been shown that PCOD is usually associated with incisors and premolars [13], and the lesions detected in the current study were most commonly associated with molars. Similarly, the radiological images of the three types of lesions (radiolucent, radiopaque and mixed) detected in this study were similar to the lesion developmental stages observed in PCOD. Nevertheless, the most significant difference between the second stage of PCOD and the mixed lesions in AS and RA is the differential location of the lytic and sclerotic areas. In conclusion, the association of lesions with different tooth groups and the different localisation of lytic and sclerotic areas show that there may not be a similarity in the development of mixed lesions between PCOS with RA and AS.

There is a clearly defined association between periodontal conditions that cause osteonecrosis in jaw bones and RA, and various hypotheses attempt to account for this epidemiological association. The most prominent hypothesis is that periodontitis is associated with and may even trigger systemic conditions and periodontal disease. Periodontal pathogens, especially porphyromonas gingivalis, may trigger autoimmunity against citrullinating proteins in the joints, thus inducing RA [16, 23]. Another hypothesis is that periodontal diseases have been shown to lead to the production of RA-related autoantibodiesthat can be detected years before the onset of RA symptoms [4]. The association between AS and periodontal diseases is supported by the detection of pronounced increases in serum interleukin (IL)-6 and tumor necrosis factor-alpha (TNF-α) levels between the immunopathological state in AS and periodontitis [24, 25]. Although the association between periodontal diseases and RA and AS is clear, few studies have investigated the association between these diseases and apical lesions that cause resorption in the jaw bone around the roots. In the literature on RA, Jalali et al. [20] found no differences between the RA and C groups in terms of the presence of apical lesions, while Karatas et al. [18] reported that patients with RA might be prone to developing apical lesions. The only study evaluating the association of AS with apical lesion development found that patients with AS disease may be inclined to develop apical lesions [19]. In these studies, clinical symptoms and the presence of radiolucent lesions were evaluated as indicators of endodontic disease [18–20]. In the current study, lesion types and prevalence were evaluated. According to the results of this research, both diseases were found to be more prone to the formation of radiopaque lesions than the C group. In contrast, the presence of radiolucent lesions in all groups was similar. It is difficult to compare the other studies’ results with those of the current study because the previous ones did not mention the presence of radiopaque and mixed lesions. In addition, the literature review showed that the relevant studies examined the association between apical periodontitis and one disease involving the body’s bones (i.e., IBD). Similar to other studies [18–20], since only radiolucent lesions were mentioned in this study, it is difficult to compare them with this study [14].

Many diseases that cause radiopaque/mixed lesions of the jaw bones have now taken their place in the literature, with renal dystrophy (RD) being one of them [9]. RD (osteitis fibrosis) is a bone and mineral pathology known to cause mixed lesions of jaw bones that result in disruptions/deficiency in the vitamin D metabolism of the body (calcitriol) and secondary hyperparathyroidism [9]. RD [9], RA and AS [3] have been associated with lower vitamin D serum levels, and RD is widely reported to result in the formation of mixed lesions [9]. However, the present study revealed the effect of RA on the formation of such lesions. This finding suggests that mixed lesions seen in patients with RA may develop through a mechanism like that of mixed lesions caused by renal dysplasia, which causes disruptions/deficiency in the body’s vitamin D metabolism. Nevertheless, AS, like RA and RD, is known to be associated with vitamin D deficiency. Further research is necessary to explain why these three diseases appear to vary in terms of the types of associated lesions. At the same time, RA and RD are characterised by mixed lesions, and radiopaque lesions are frequently observed in AS. The literature review showed that chronic kidney disease (CKD) is common in patients with RA, and its prevalence ranges from 37 to 57% [26]. Conversely, the literature review on studies addressing the association between AS and renal diseases did not yield any publications pointing to a direct association. However, one review of systemic amyloidosis established RA as a direct factor in the development of secondary amyloidosis [27]. Regarding AS, one study in the literature found secondary amyloidosis affecting the kidneys and diagnosed at an advanced stage in 8 (1.1%) out of 730 patients with AS [28]. The fact that the incidence of mixed lesions in patients with AS is not significant may be related to the less common occurrence of secondary amyloidosis affecting the kidneys in patients with AS. This supports our argument that mixed lesions in RD [9] and RA patients with common CKD [26] may have a similar developmental mechanism. We also assume that increased osteoblastic activity may represent the mechanism of radiopaque lesions seen in patients with AS, such as the mechanism of spinal ankylosis, which can also be observed in these patients [8, 20].

RA and AS diseases are known to follow a similar clinical course in terms of symptoms, radiographic characteristics and serological testing [4]. However, disease-modifying antirheumatic drugs known to be effective in the treatment of RA have unfortunately failed in the treatment of AS [2]. For example, methotrexate, which is used to improve and delay symptoms in patients with RA, may not be effective in patients with AS [2, 5]. On the contrary, there is some evidence that anti-TNF treatments (TNF blockers) [6] and, similarly, IL-17 blockers [7] are more effective in the treatment of AS than RA. Another interesting finding comes from Karatas et al. [19], who demonstrated that apical lesions healed faster in patients on anti-TNF-α than in their C group.

The effect of anti-citrullinated protein antibodies (ACPAs) and rheumatoid factor (RF) autoantibodies produced in RA patients on bone resorption was stated [29, 30]. However, some mediators, such as TNF, IL-1 and IL-7, stimulate osteoclast transformation from osteoclast precursors [31–33]. Anti-TNF agents and IL-6R antagonists used in treatment have an inhibiting effect on bone destruction [34].

Rheumatological diseases, including AS and juvenile spondyloarthritis, are defined as seronegative because, unlike RA patients, RF or ACPAs are not observed [35]. For this reason, the possible bone-destroying effects of these autoantibodies do not occur in AS patients.

A remarkable mechanism regarding bone formation has been mentioned in patients with AS. Yang et al. stated that hypertrophic chondrocytes can acquire osteogenic properties after the embryonic period and can exist until advanced ages. This situation, defined as ‘chondrocyte–osteoblast lineage continuity’, and AS [36] may be linked to events that lead to new bone formation. Sclerostin is another component involved in bone remodelling in both AS and RA [37].

This study aimed to offer a retrospective evaluation based on DPRs for the types and prevalence of periapical lesions in patients with RA or AS. In this respect, the study’s limitations are the lack of datas as the vitamin D levels of patients with AS or RA and their medication and treatment or lab confirmation, as well as further information on pain, vitality, percussion and palpation sensitivity recorded during an oral clinical examination. We also lacked information indicating whether the lesions observed in the teeth with RCT presented a progressive or regressive infection. Additionally, the use of DPRs to save patients from unnecessary radiation has created a significant limitation, especially for detecting apical lesions in the anterior region. Finally, the use of a randomised C group with a proportionate number of subjects to that of all eligible patients with AS and RA revealed a significant difference among the groups in terms of gender, age and the total number of teeth. To overcome this limitation, multivariate regression analyses were applied, and adjusted ORs were given to eliminate this issue.

The present study evaluated the possible correlation of the incidence of radiolucent, radiopaque and mixed lesions with RA and AS diseases. According to the results, AS and RA may either completely or partially induce increased bone density and thus radiopacity. Clinicians must be aware of the prognosis of periradicular pathologies for these patients, which may be represented as dense bone formation. Furthermore, regarding the risk of ankylosis resulting from this dense bone, the extraction of the relevant teeth of these patients may be complicated.

## Conclusion

The results of this study revealed that further studies should examine patients with AS or RA with a complete dataset, including data on their treatment and medication (i.e. vitamin D levels and use of anti-TNF-α, IL-6R antagonists, methotrexate etc.), clinical oral examination (i.e. source of infection, pain, vitality of involved tooth, percussion/palpation sensitivity and history of dental treatment) and radiological examination (i.e. presence and types of lesions). Further studies evaluating this complete dataset may account for similarities between the two diseases in terms of clinical processes and affected tissues and the reason for the common occurrence of radiopaque lesions in both patient groups in the face of the common detection of mixed lesions only in patients with RA.

## Data Availability

The data that support the findings of this study are available from the corresponding author upon reasonable request.

## Abbreviations

DPRs: Dental digital panoramic
RA: Rheumatoid arthritis
AS: Ankylosing spondylitis
RCT: Root canal treatment
IBD: Inflammatory bowel disease
PCOD: Periapical cemento-osseous dysplasia
